# Supplementary material for: Deep learning-based automatic action extraction from structured chemical synthesis procedures
Source: PeerJ Comput Sci. 2023 Aug 18;9:e1511. doi: 10.7717/peerj-cs.1511 (PMC10495970; doi:10.7717/peerj-cs.1511)
Supplement: Supplemental Information 1 — The action names and the associated format. The bold words in the “Format” column appear in the text as they are, while the time, chemical name, or temperature replace other words. Certain parameters may not be present in the original text; therefore, such parameters are omitted from the structure format. The table describes the full version of the action. [file peerj-cs-09-1511-s001.docx]

**Appendix S1**

Appendix S1 table 1 describes the action names and the associated format. The bold words in the “*Format*” column appear in the text as they are, while the time, chemical name, or temperature replace other words. Certain parameters may not be present in the original text; therefore, such parameters are omitted from the structure format. The table below describes the full version of the action.

Table 1. Action names and the format.

| **Action name** | **Format** |
| --- | --- |
| InvalidAction | ***INVALIDACTION*** |
| Add | ***ADD*** *chemical name* ***(****quantity****)*** ***at*** *temperature* ***under*** *chemical name* ***over*** *time* |
| CollectLayer | ***COLLECTLAYER*** *(aqueous/organic)* |
| Concentrate | ***CONCENTRATE*** |
| Degas | ***DEGAS*** ***with*** *chemical name* ***for*** *time* |
| DrySolid | ***DRYSOLID*** ***for*** *time* ***at*** *temperature* ***under*** *atmosphere* |
| DrySolution | ***DRYSOLUTION*** ***over*** *chemical* |
| Extract | ***EXTRACT*** ***with*** *chemical name number* ***x*** |
| Filter | ***FILTER*** ***keep*** *phase* |
| FollowOtherProcedure | ***FOLLOWOTHERPROCEDURE*** |
| MakeSolution | ***MAKESOLUTION*** ***with*** *chemical name* ***(****quantity****)*** ***and*** *chemical name* ***(****quantity****)*** |
| Microwave | ***MICROWAVE*** ***for*** *time* ***at*** *temperature* |
| OtherLanguage | ***OTHERLANGUAGE*** |
| Partition | ***PARTITION*** ***with*** *chemical name* ***and*** *chemical name* |
| PhaseSeparation | ***PHASESEPARATION*** |
| Purify | ***PURIFY*** *gradient ratio-ratio chemical name:chemical name* |
| PH | ***PH*** ***with*** *chemical name* ***to PH*** *number* |
| Quench | ***QUENCH*** ***with*** *chemical name* ***at*** *temperature* |
| Recrystallize | ***RECRYSTALLIZE*** ***from*** *chemical name* |
| Reflux | ***REFLUX for*** *time* ***at*** *temperature* ***under*** *chemical name* |
| SetTemperature | ***SETTEMPERATURE*** *temperature* |
| Sonicate | ***SONICATE*** ***for*** *time* ***at*** *temperature* |
| Stir | ***STIR*** ***for*** *time* ***at*** *temperature* ***under*** *chemical name* |
| Triturate | ***TRITURATE*** ***with*** *chemical name* |
| Wait | ***WAIT*** ***for*** *duration* ***at*** *temperature* |
| Wash | ***WASH*** ***with*** *chemical name number* ***x*** |
| Yield | ***YIELD*** *chemical name* |
| NoAction | ***NOACTION*** |
